# Climate Change and Goat Production: Enteric Methane Emission and Its Mitigation

**DOI:** 10.3390/ani8120235

**Published:** 2018-12-07

**Authors:** Pratap Pragna, Surinder S. Chauhan, Veerasamy Sejian, Brian J. Leury, Frank R. Dunshea

**Affiliations:** 1 ICAR—National Institute of Animal Nutrition and Physiology, Adugodi, Bangalore 560030, India; pragnaprathap@gmail.com; 2 Academy of Climate Change Education and Research, Kerala Agricultural University, Vellanikkara 680656, India; 3 Faculty of Veterinary and Agricultural Sciences, The University of Melbourne, Parkville, VIC 3010, Australia; ss.chauhan@unimelb.edu.au (S.S.C.); brianjl@unimelb.edu.au (B.J.L.); fdunshea@unimelb.edu.au (F.R.D.)

**Keywords:** climate change, heat stress, goat, immunization, methane, volatile fatty acids

## Abstract

**Simple Summary:**

Given that goats are considered more climate resilient than other ruminant species, research efforts are therefore needed to understand goat productivity during exposure to high ambient temperatures. Heat stress can affect the digestion and rumen fermentation pattern of goats, which contributes to the reduction in production performance in goats. Diet composition, breed and environmental stresses are common factors which negatively influence rumen function and enteric methane (CH_4_) emission. There are three mechanisms by which enteric CH_4_ can be reduced: targeting end product of digestion to propionate, providing alternate hydrogen sink and selectively inactivating rumen methanogens. The various strategies that can be implemented to mitigate enteric CH_4_ include nutritional interventions, management strategies and application of advanced biotechnological tools.

**Abstract:**

The ability of an animal to cope and adapt itself to the changing climate virtually depends on the function of rumen and rumen inhabitants such as bacteria, protozoa, fungi, virus and archaea. Elevated ambient temperature during the summer months can have a significant influence on the basic physiology of the rumen, thereby affecting the nutritional status of the animals. Rumen volatile fatty acid (VFA) production decreases under conditions of extreme heat. Growing recent evidence suggests there are genetic variations among breeds of goats in the impact of heat stress on rumen fermentation pattern and VFA production. Most of the effects of heat stress on rumen fermentation and enteric methane (CH_4_) emission are attributed to differences in the rumen microbial population. Heat stress-induced rumen function impairment is mainly associated with an increase in *Streptococcus* genus bacteria and with a decrease in the bacteria of *Fibrobactor* genus. Apart from its major role in global warming and greenhouse effect, enteric CH_4_ is also considered as a dietary energy loss in goats. These effects warrant mitigating against CH_4_ production to ensure optimum economic return from goat farming as well as to reduce the impact on global warming as CH_4_ is one of the more potent greenhouse gases (GHG). The various strategies that can be implemented to mitigate enteric CH_4_ emission include nutritional interventions, different management strategies and applying advanced biotechnological tools to find solution to reduce CH_4_ production. Through these advanced technologies, it is possible to identify genetically superior animals with less CH_4_ production per unit feed intake. These efforts can help the farming community to sustain goat production in the changing climate scenario.

## 1. Introduction

Morphologically versatile goat species with unique browsing potential adapt to a changing climate more readily than other ruminant species and consequently they continue to be an important source of income and nutrition to many poor and marginal farmers around the world [1]. Goats are also the major means of employment and income for women, children and aged people in tropical and subtropical regions [2]. The important sources of income from the sector include milk, meat, manure, wool and skin [3]. Small ruminant, and in particular goat, farming is very important because of the relatively low input requirements and the corresponding high expected output [4]. Furthermore, goats emit less enteric methane (CH_4_) than all other domestic ruminant animals per unit body weight [5].

A changing climate scenario for extensive grazing systems exposes the animals to various types of stressors that may affect their production, health and survival [6]. Among these, heat stress seems to be the major stressor which negatively influences the animal performance [7]. Furthermore, heat stress can also affect the digestion and rumen fermentation pattern of goats which contributes to the reduction in production performance [8]. The ability of an animal to cope and adapt itself to a changing climate depends on maintaining appropriate functioning of the rumen and ruminal microbes [9]. Elevated ambient temperature may prove detrimental to these processes and may ultimately result in influencing the level of CH_4_ production particularly with respect to the intensity of its production in goat and this will require appropriate mitigation strategies to curtail such emissions to sustain goat production in the changing climate scenario [8]. Given that goats are considered more climate resilient than other ruminant species, research efforts are therefore needed to understand goat productivity during exposure to high ambient temperatures. This review is therefore an attempt to collate and synthesize existing knowledge and recent research pertaining to the effects of heat stress on rumen fermentation, enteric CH_4_ emissions, and the various mechanisms associated with CH_4_ production and its mitigation in goats. 

## 2. Goat as Ideal Climate Model Animal

Small ruminants, in particular goats, are considered an important source of income and nutrition for poor and marginal farmers around the world [5]. Low initial investment and high turnover rate for goat production are the primary reasons behind the promotion of the goat industry in developing countries [10]. Goats are often referred to as village banks in some rural areas where the villagers invest their money on purchasing and feeding goats and consider it as an appropriate way to save money for the future [11]. Globally, there are estimated to be over 860 million goats [12] and recent trends show an increased demand for dairy products from goats, particularly in developing countries where they act as a substitute for dairy products from large ruminants for human dietary needs [13]. 

Goats are versatile animals that adapt to a changing climate more readily than the other ruminant species and are well suited to small farming systems [1]. Much of the global goat population is concentrated in the arid and semi-arid agro-ecological zones that have frequent droughts and famines [14]. However, these species are reported to be less affected by the harsh climate compared with other ruminants that are highly sensitive to subtle changes in the surrounding environmental fluctuations [15]. Hence, goat rearing is a major source of human nutrition and also the means of economic stability for many small and marginal farmers, providing meat and manure as two major sources of income [14]. 

Because of their browsing habit and the anatomical advantage of the upper lips, goats can thrive well with limited feedstuffs, especially in arid and semi-arid regions [16]. In addition, goats also have a physiological advantage because they efficiently utilize poor quality feedstuffs and produce appreciably good output in terms of milk, meat and manure [17]. During feed scarcity, goats can reduce their metabolic processes to conserve energy resources [8]. Table 1 describes the advantageous characteristics in goats over other livestock species to survive harsh climatic conditions.

## 3. Impact of Heat Stress on Rumen Function

Elevated ambient temperature during the summer months can have a significant influence on the basic physiology of rumen function, thereby affecting the nutritional status of the animals [23]. Rumen volatile fatty acid (VFA) production is altered during the conditions of extreme temperature, while feed digestibility is increased with increasing ambient temperature because of a reduction in feed intake and passage rate, which allows more time for the microbes and enzymes to digest feed [24]. Table 2 describes the various impacts of heat stress on rumen function.

### 3.1. Rumen Fermentation Pattern

Environmental factors such as temperature and relative humidity (RH) can have significant role in the feed consumption of animals. An increase in temperature and RH decreases the dry matter intake of the animals and rumination as a result of increased amount of buffering agents entering the rumen and this could be attributed to the reduced chewing activity [7]. Additionally, blood flow is redirected from the gastrointestinal tract to the periphery for heat dissipation, which further decreases the digestibility [8]. Furthermore, an increased respiration rate during summer season increases expired CO_2_ output leading to decreased blood and rumen pH and acidosis [25]. Likewise, Castro-Costa et al. [26] reported a reduction in ruminal pH in heat exposed Murciano-Granadina dairy goats and attributed this to the reduced rumen fermentation during heat stress. Similarly, Yan-fen et al. [27] also reported a decreased rumen pH and NH_3_-N concentration in dairy goats exposed to heat stress. 

### 3.2. Volatile Fatty Acid Production

There are reports showing a decrease in VFA production during the periods of heat stress [28,29]. Similarly, Tajima et al. [30] reported a decrease in acetate and acetate to propionate ratio and an increase in butyrate level in heat stressed animals, which they attributed to alterations in the number of rumen microbiota during the periods of heat stress. Likewise, Hirayama et al. [24] reported a reduction in plasma acetate and VFA concentrations in heat exposed (35 °C) Saanen goats compared to Saanen goats kept under thermoneutral conditions (20 °C). They attributed these changes to reduced feed intake and rumen microbial diversity. Further, in a study conducted in indigenous goat breeds, we [28] reported a reduced production of acetate concentrations in heat exposed Osmanabadi and Malabari goats, whereas the Salem goats did not exhibit any change. In the same experiment, we also observed an increase in propionate concentration in the Salem black goats and a decline in the propionate production in Malabari goats. These variations in the heat stress response could be explained by the differences in the adaptive capability among the breeds, suggesting Salem black as the superior adaptive breed in the climate change scenario. Further, Chaidanya et al. [29] reported a reduction in VFA concentrations in rumen of goats exposed to high ambient temperature coupled with high relative humidity. The reduction in the VFA concentration could be attributed to the increased rumen temperature during the heat stress periods. 

### 3.3. Rumen Microbial Population

Heat stress induced rumen function impairment is mainly associated with an increase of *Streptococcus* genus bacteria and a decrease in the bacteria of *Fibrobactor* genus [31]. Further, Tajima et al. [30] also reported these changes along with altered rumen bacterial diversity with a decrease in uncultivated Cluster E group sequences during heat stress. Similarly, Uyeno et al. [32] observed a decrease in the *Streptococcus* genus and an increase in both *Streptococcus* spp. and *Clostridium coccoides–Eubacterium* genus in the rumen. Changes in the rumen microbial ecosystem due to heat exposure can influence feed digestibility and composition of the end products by altering the rumen fermentation pattern [32]. Further, Bernabucci et al. [9] observed a decline in the concentrations of amylolytic and cellulolytic bacteria in animals exposed to ambient conditions having a temperature humidity index (THI) 85. The decreased dry matter intake and passage rate in heat stressed animals could reduce the bacterial diversity ultimately culminating in decreased diet digestibility [9]. There are few research reports available on how high ambient temperature selectively affects microbial population. However, this impact could be attributed to the sensitivity of certain rumen microbes to increased temperature exposure.

### 3.4. Enteric Methane Emission

**Figure 1:**
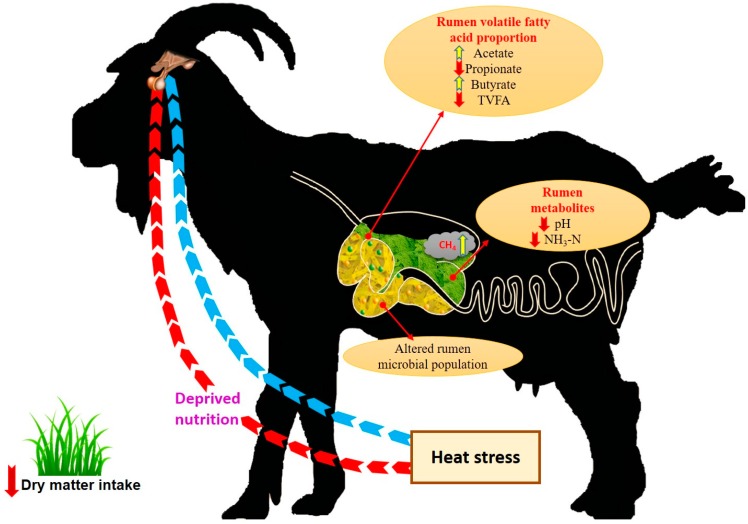
Impact of heat stress on various rumen functions in goat (these concepts were adopted from References [28,29]). TVFA: Total Volatile Fatty Acid.

Environmental temperature is a key factor that determines CH_4_ production, since feed intake and digestibility differ with ambient temperature. Mbanzamihigo et al. [33] reported an increase in enteric CH_4_ emissions during late summer (August–September) compared to early summer (June–July) in the Northern Hemisphere. Similarly, in another experiment conducted in young wethers grazing a moist hilly island pasture, a perennial rye grass/white clover dominant pasture and a late summer season pasture showed CH_4_ yields of 4.1%, 3.9% and 5.3%, respectively. Increased CH_4_ yield in wethers grazing late summer season pastures is attributed to the quality deterioration (poor dry matter digestibility, lower protein and soluble carbohydrate content and increased cell wall content) of the pastures during the summer season [34]. This study revealed the indirect effect of elevated ambient temperature on the CH_4_ production through altered pasture characteristics. Further, Ulyatt et al. [35] reported an increase in CH_4_ emission during grazing of summer grassland compared to Kikuyu grassland. Figure 1 shows the impact of heat stress on various rumen functions in goat.

### 3.5. Factors Influencing Enteric Methane Emission in Goats

Various factors affect the enteric methane production in goats and these are broadly classified as weather associated factors such season and increased ambient temperature; feed associated factors such as diet composition, time after feeding, and feed additives; and animal associated factors that include inflow of saliva, types of microbial population, and breed [36].

Composition of feed is the primary factor that determines the rumen fermentation pattern and enteric methane emissions [37]. Further, the propionate to acetate ratio also influences the rumen fermentation pattern and is determined by the concentrate to forage content of the diets [38]. In comparison with roughage feed, concentrates contain less structural carbohydrates, so the intake of concentrates may increase the production of propionate and decrease the production of acetate, ultimately resulting in reduced CH_4_ production. An increase in concentrate intake is associated with increased propionate production and this may reduce the number of H_2_ atoms available to the methanogenic bacteria, again resulting in reduced methane production. However, the higher level of concentrate feeding can cause sub-acute acidosis, both sub-clinical and clinical, which may adversely impact normal ruminal fermentation processes through both alteration of the functions of essential rumen microbes and impaired VFA absorption due to low ruminal pH [39].

In recent years, the usage of microbial feed additives has increased to improve growth performance of meat animals. In addition, some microbial feed additives have been used to reduce CH_4_ production in ruminant animals. Malik et al. [40] used acetogens as a feed additive to replace prominent CH_4_-producing methanogenic bacteria to reduce enteric methane production by acting as alternate hydrogen. The prominent CH_4_-producing methanogenic bacteria have a low H_2_ threshold level, thus do not allow the naturally resident acetogens to utilize hydrogen. Other feed additives such as fat and oil supplements have also been reported to have an effect on the rumen fermentation profile, thereby reducing rumen protozoan population and CH_4_ reduction [41]. However, high fat diets can alter the rumen microbial population and ultimately it can hamper the fibre digestibility by specifically inactivating the rumen microbes that are associated with fibre digestion [41]. Plant bioactives, including saponins and tannins, can reduce CH_4_ production in ruminants [42].

Breed is another important factor that determines enteric CH_4_ production [43]. These breed-to-breed differences in enteric CH_4_ production could be attributed to their variation in body size, adaptation, rumen volume and the variation in the feed intake [43]. Rumen associated factors such as rumen pH, type of volatile fatty acids fermented, type of substrates fermented, rate of fermentation, absorption capacity of rumen wall, and rumen protozoa concentration determine the level of CH_4_ production [44]. Rumen methanogens remove H_2_ molecules that are synthesized during the organic matter fermentation produced during fermentation of organic matter in the hind gut and rumen and produce CH_4_ [45]. Further, the increased production of propionate decreases the CH_4_ production by consuming H_2_ molecules [46].

**Figure 2:**
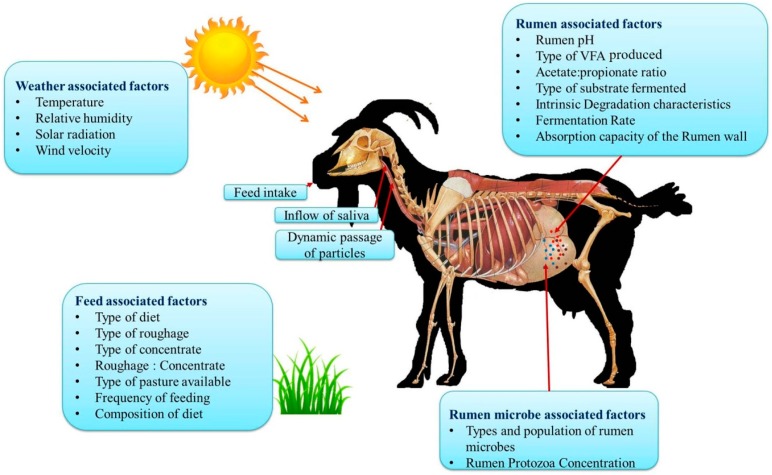
Various factors influencing enteric methane emission in goats (these concepts were adopted from References [8,28,29]).

Geographic location and climate are known to be the most crucial factors significantly affecting CH_4_ production and this could be due to ambient temperature differences as well as difference in feed resources available [44]. Animals reared in arid and semi-arid regions have been reported to produce less CH_4_ production compared with animals in temperate regions, and this could be due to the differences in the type or amount of feed consumed in different locations [44]. Among the climate variables temperature, humidity, solar radiation and wind velocity are the important variables that influences CH_4_ production. Increased ambient temperature coupled with high relative humidity (RH) directly affects CH_4_ production by altering the rumen fermentation profile and indirectly by altering the quality of pasture or forage [46]. Although heat stress may reduce the feed intake, the increased methane emission could still be attributed to the heat stress associated negative impact on feed digestibility by inhibiting the rumen microbial populations that are essential for the normal digestion process. The various factors influencing enteric methane production from goats are summarized in Figure 2.

### 3.6. Enteric Methane Mitigation Strategies in Goats

**Figure 3:**
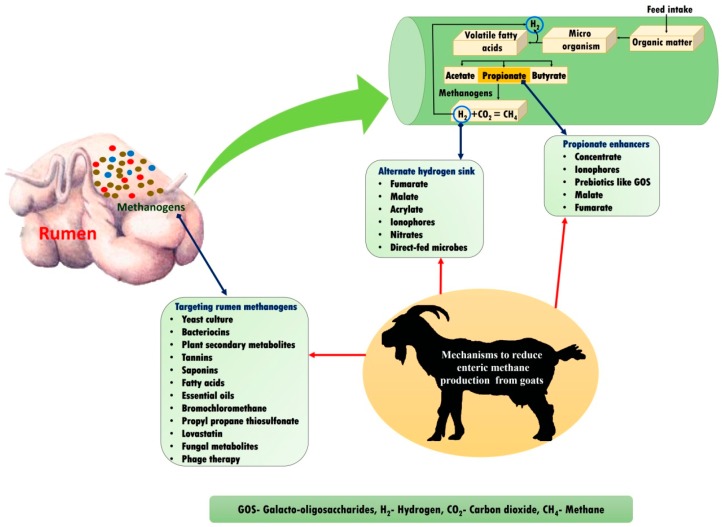
Various mechanisms to reduce enteric methane emission in goats.

Apart from its major role in global warming and the greenhouse effect, enteric CH_4_ is also considered as a dietary energy loss of around 2–12% in ruminants. Consequently, the global scientific community is targeting the development of suitable CH_4_ mitigation strategies to reduce both global warming and dietary energy loss. The various strategies that can be implemented to mitigate enteric CH_4_ include feeding feed sources containing plant secondary metabolites, ration manipulation, fat and oil supplementation, bacteriocin supplementation, rumen modification, etc. [29,45,46,47]. Various mechanisms to reduce enteric methane production in goats are summarised in Figure 3.

#### 3.6.1. Nutritional Intervention to Reduce Enteric Methane Production in Goats

Among the various CH_4_ mitigation strategies, nutritional intervention or dietary manipulation is the most effective and commonly used strategy to mitigate enteric CH_4_ emission in ruminant livestock [48,49]. It is well known that increasing the ratio of concentrate to forage in the diet can reduce the amount of energy loss as enteric CH_4_ and this is mainly due to change of fermented substrate from fibre to starch [48]. In an experiment conducted on Murciano-Granadina goats in late lactation, Ibáñez et al. [50] observed a lower CH_4_ production in goats fed with concentrate (ground corn) diet than beet pulp fed goats. However, concentrate feeding beyond a certain limit is not appreciable as it can cause severe damage to the animal itself and to its production performance. In addition, grains that may be used for concentrates are more valuable for human feeds in arid and semi-arid regions where much of the global goat production is located.

Supplementing the feed with more lipids and fatty acids was reported to reduce the dietary energy loss in goats [51]. However, the effectiveness of lipid supplementation relies on the source, inclusion rate, fatty acid profile and the composition of the rest of the diet [48,52]. Reduction in enteric CH_4_ emission to the tune of around 40% is possible using high quality lipid feed supplements [46]. By differing the mode of action, lipid feed additives may reduce the methanogen and ciliated protozoan population in the rumen. Further, lipid supplementation reduces fibre and organic matter degradability and decreases the fermentable substrate availability and thereby minimising CH_4_ production [53]. Abubakr et al. [54] conducted an experiment in Boer X Catcang crossbred goats where they found that adding decanter cake and palm kernel cake at up to 80% inclusion decreases methanogenesis by reducing rumen protozoa in goats. Further, Zhou et al. [55] reported the ability of lauric acid to reduce CH_4_ production by reducing the viability of *Methanobrevibacter ruminantium*. Likewise, Kong et al. [56] reported a significant reduction in the methanogenesis without affecting the quantity of rumen methanogenic archaea after flaxseed supplementation.

Ionophore supplementation is another extensively researched CH_4_ abatement strategy. Ionophores cause a shift in the rumen fermentation pattern from acetate and butyrate production to propionate by increasing the gram-positive bacteria population, resulting in decreasing the production of CH_4_ [57]. Monensin is the most studied ionophore and routinely used as an animal nutrition supplement [58]. Saanen goats supplemented with oils with sodium bicarbonate and monensin showed a shift in the production of molar concentrations of acetate to propionate, thereby reducing the production of CH_4_ [59]. Furthermore, up to a 75% reduction in CH_4_ production was observed on addition of 10% encapsulated fumarate to the diet without any negative effect on animal growth [58]. 

Although several anti-methonogenic compounds are well proved in terms of their CH_4_ reduction potential, certain individual components have antinutritional properties that inhibit their commercial usage. However, data obtained from anti-methonogenic supplementation studies are good models and they can pave a way towards effective CH_4_ mitigation strategies [60,61]. Abecia et al. [45] conducted an experiment in Murciano-Granadina lactating goats to evaluate the potential of bromochloromethane (BCM) complex to reduce enteric CH_4_ production and they observed 32% reduction in BCM fed goats as compared to the control group. In another experiment conducted in Murciano-Granadina goats, Martínez-Fernández et al. [62] observed 33% and 64% methane reduction per kg of dry matter intake with propyl propane thiosulfinate (PTS) and BCM supplementation, respectively. Further, Murciano–Granadina goats supplemented with PTS and BCM decreased CH_4_ production by 48% and 98%, respectively, which was attributed to the redirection of H_2_ from CH_4_ production to propionate metabolic pathways [63]. Similarly, Mitsumori et al. [64] also reported 71% and 91% reductions in CH_4_ production in Shiba Japanese goats supplemented with 2 g/100 kg Live Weight and 5 g/100 kg LW of BCM, respectively. Candyrine et al. [65] conducted a study on Saanen goats with three levels of lovastatin (naturally produced from fermentation of palm kernel with *Aspergillus terreus*) supplementation and the authors observed 7.8%, 20% and 21% CH_4_ reduction for low (2 mg lovastatin/kg BW/day), medium (4 mg lovastatin/kg BW/day) and high (6 mg lovastatin/kg BW/day) treatment groups, respectively. Further, Azlan et al. [66] reported 32% reduction in enteric CH_4_ production when supplementing Boer crossbred goats with 14 mg/kg BW of lovastatin produced from rice straw treated with *Aspergillus terreus*.

Microbial feed additives are another important nutritional intervention in the CH_4_ mitigation studies. Apart from the effects on CH_4_ mitigation, probiotic feeding can improve the growth performance of meat animals and it can also reduce the incidence of diarrhoea [67]. However, studies proving the efficiency of direct fed probiotics to reduce the production of enteric CH_4_ are few [68]. The same authors also reported that nitrate as feed additive can reduce rumen methanogenesis in different ruminant species and production conditions [68]. Chaucheyras-Durand et al. [69] showed that yeast cells can reduce the production of enteric CH_4_ by deviating hydrogen atoms from methanogens to acetogenic strains of ruminal bacteria to enhance the production of acetate. Yeasts such as *S. cerevisiae* and the lactic acid utilizing bacteria *Propionibacterium* spp. and *Megasphaera elsdenii* can decrease rumen methanogenesis when included in the diet as supplements [60]. Wang et al. [61] found that replacing ordinary rice feed with red yeast rice, which is a traditional Chinese culinary and medicinal product, resulted in a 13% reduction in CH_4_/DM intake in Boer crossbred goats.

Organic acids such as malic acid and fumerate have the potential to reduce CH_4_ production in the ruminant by serving as an alternative hydrogen sink. Organic acid administration has been proven to reduce methane production in a dose-dependent manner in several in vitro studies [70]. In an experiment conducted in Xinong Saanen dairy goats, Li et al. [71] reported a significant reduction in CH_4_ production in goats supplemented with fumaric acid. Further, in the same study, along with fumeric acid supplementation, the authors also altered the particle size of concentrate and forage feed and observed 32% and 18% CH_4_ reduction in low forage and concentrate particle size diet and high forage and concentrate particle size diet, respectively [71].

Phenolic monomers, condensed tannins and other plant secondary metabolites in dose-dependent manner can reduce enteric CH_4_ emission from the ruminants because of their ability to reduce methanogenesis. Puchala et al. [72] reported 57% reduction in CH_4_ in terms of g/kg DMI in condensed tannin containing *Lespedeza cuneata* fed Angora goats compared to Angora goats fed a combination of *Festucaarundinacea* and *Digitariaischaemum*. Dietary tannins can directly hinder CH_4_ production as well as indirectly limit methanogenesis through reducing the availability of hydrogen atoms. In a meta-analysis using 30 experiments comprising 171 treatments to evaluate the extent of dietary tannins to reduce the CH_4_ emission, Jayanegara et al. [73] found a negative correlation between enteric CH_4_ production and tannin supplementation. Furthermore, Wina et al. [74] reported a reduction in methanogens in methanol extract saponin containing *Sapindus rarak* fed animals. Similarly, Mao et al. [75] reported a 27% reduction in enteric CH_4_ production with tea saponin supplementation. Further, in an experiment conducted in goats fed with natural tannin containing *Mimosa* spp., Bhatta et al. [46] reported a CH_4_ reduction after *Mimosa* spp. supplementation even at low concentrations (2–8 g/kg DM of the diet). In a study conducted in Nanjiang Yellow goats, Dong et al. [76] reported a reduction in enteric methane production on *Artemisiae annuae* extract and herbal medicines mixture supplementation to different diets. Further, under in vitro condition, Denman et al. [77] reported 91% reduction in methane production using bromochloromethane at 5 g/100 kg LW in Japanese native goats. 

#### 3.6.2. Management Strategies to Reduce CH_4_ Production from Goats

Improving management strategies not only reduces enteric methane emission but also helps to improve animal productivity [78]. Reduction or culling of unproductive animals from the herd has the potential to simultaneously improve the productivity and to reduce CH_4_ emission [79,80]. In subsistence production systems, reduction in the herd size allows distribution of adequate amount of feed and proper veterinary care to all animals. Additionally, selective culling can reduce CH_4_ production both per unit of animal product and for the total herd [81]. However, in some subsistence farming systems, there may be insufficient high breeding value animals to allow selective culling. Slaughter weight of goats can be advanced at a young age through early finishing approaches. This can potentially reduce the lifetime net CH_4_ emissions, thus making available proportionally few CH_4_ producing animals [79]. 

Reductions in enteric CH_4_ production can be achieved through efficient pasture management practices in goats. Feeding animals high quality fodder can reduce the wastage of dietary energy. Improving quality of the forage also increases feed intake and reduces the retention time of digesta in the rumen, thereby stimulating energetically more efficient post-ruminal digestion and decreases the percentage of energy transformed to CH_4_ [79]. Sejian et al. [82] reported a reduced CH_4_ emission in animals fed with high quality fodder as compared to animals consuming low quality fodder. Reductions in enteric CH_4_ production can also be achieved through feeding high quality fodder with higher soluble carbohydrates and lower fibre or through grazing on less-matured pastures [48,79]. Harvesting or grazing of forage at early stages of maturity also reduces the plant cell wall lignification, thereby increasing digestibility and reducing the CH_4_ emission per unit of digestible dry matter [83]. Similarly, Pinares-Patiño et al. [84] conducted a grazing experiment in timothy pasture at four different vegetative phases, namely, early vegetative stage, heading, flowering and senescence, and they observed lower CH_4_ production only at heading stage, which confirms the significance of growth stages of forage in CH_4_ production. Waghorn and Hegarty [85] calculated that animals grazing on high quality pasture (20% higher ME value) may show a reduction in enteric CH_4_ production of approximately 50%. Likewise, animals consuming certain high quality tropical and temperate legumes show reduced enteric CH_4_ production, as the legumes contain condensed tannins that are toxic to methanogenic archaea, ciliate protozoa, and fibre degrading bacteria [86]. Further, the grasses with high concentrations of water-soluble carbohydrates have been investigated as suitable tool to reduce enteric CH_4_ emission from the ruminant livestock [87]. De Ramus et al. [88] reported 22% reduction in enteric CH_4_ production annually through the efficient use of grazed forage crops through management-intensive grazing. Furthermore, around 5% reduction in enteric CH_4_ production is possible through improving total tract NDF digestibility [53]. Archimede et al. [89] reported 17% more CH_4_ production from animals fed with C4 grasses than the animals fed with C3 grasses.

In the majority of regions around the globe, goats are raised under continuous grazing systems, where animals have ad libitum access to pasture. However, unrestricted access to the pasture can result in the elimination of edible pasture and the domination of less edible pasture due to the uncontrolled selective grazing [31]. Hence, adoption of controlled grazing is a reliable strategy to reduce enteric CH_4_ and to improve productivity. In these systems, grazing land is divided into different paddocks that are alternatively grazed and rested until the pasture restores its quality. A continuous supply of uniform quality feed throughout the year enables animals to increase their production and to decrease CH_4_ production per kilogram of weight gain [20].

Size of the forage has profound effect on the CH_4_ emissions. Animals deviate considerable amount of their energy to the chewing process [90]. Particle size reduction of fodder by mechanical means helps to enhance digestibility through bringing more microbial access to the substrate, decreasing energy expenses, CH_4_ production and increasing the passage rate of digesta and animal productivity [91].

Selection of genetically superior animals with less CH_4_ production per unit feed intake is another management strategy that can be employed to reduce CH_4_ production from the ruminants [92]. The direct selection of low CH_4_ producing animals is practically impossible because of high cost for measuring CH_4._ However, selection is possible through the indirect means such as rumen digesta retention time and feed intake [80]. Genetic selection of goats with higher feed conversion efficiency generates a reduced amount of CH_4_. Further, genetic selection for the less CH_4_ production indirectly helps the farmer to increase their profits without any extra carbon credits by increasing the feed conversion efficiency and growth rate per animal [92]. A 3–10% reduction in CH_4_ production can be achieved through improving the feed use efficiency by 10% [93].

#### 3.6.3. Advanced Biotechnological Tools for Methane Mitigation

The inhibition of enteric CH_4_ emission in ruminant animals is possible though biotechnological interventions. One of the possible future strategies to reduce enteric CH_4_ production is to immunize the animals against their own methanogens. In an experiment conducted in Australia using vaccines against three selected methanogens, Wright et al. [94] reported 8% reduction in CH_4_ production. However, another experiment conducted in different geographical zone with vaccines prepared using different set of bacteria could not elicit any positive response [94]. The reasons for the immunization failures could be due to the variation in rumen methanogenic diversity present in the animals raised in different conditions and the replacement of the biological niche left by the targeted species by another methanogen [95]. CH_4_ inhibition was also attempted through oral supplementation IgY as a feed additive [96]. Zhang et al. [97] conducted an experiment in Boer goats to evaluate the efficiency of a candidate vaccine protein (EhaF) on the rumen methanogens and microbes but did not find any changes in CH_4_ production among control and vaccinated goats. However, vaccination influenced the composition of rumen bacteria.

Use of bacteriocins offers another possible strategy to reduce CH_4_ emission from ruminant animals. Bacteriocins are the proteins produced by bacteria that can obstruct certain microbial species in the rumen [98]. An in vitro study conducted by Lee et al. [99] using bovicin HC5 (a bacteriocin produced by *Streptococcus* spp.) showed 50% reduction in CH_4_ production without inducing methanogen adaptation. Likewise, Santoso et al. [100] reported a 10% reduction in CH_4_ emission in an in vivo study that used nisin, a bacteriocin produced by *Lactobacillus lactis* subsp.

The lytic potential and genes of the bacteriophages makes them potential tools to mitigate enteric CH_4_ emission [101]. Certain bacteriophages may inhabit the rumen wall to maintain the homeostasis of the rumen micro fauna. Due to their host specific nature, they lyse certain microbes such as methanogens and *Streptococcus bovis* or pathogens such as *salmonella* and *E. coli* O157:H7 [47]. McAllister and Newbold [70] reported that siphophages (*Siphoviridae* phage) can infect methanogens such as *Methano brevibacter*, *Methanobacterium* and *Methanococcus* spp.; however, siphophages have yet to be isolated from the rumen. However, there are few available data relating to the genetic functionality and blueprint of the archaeal methanogenic phages and, to date, no bacteriophages from rumen have been isolated [47].

Another plausible method of biological control of methanogens is the use of CH_4_ oxidizers. The CH_4_ oxidizing bacteria have already been isolated from the rumen [102]. However, in vitro studies conducted using carbon isotopes reveal that only 0.3–8% of CH_4_ oxidation to CO_2_ happens in the rumen [103]. Valdez et al. [104] reported a reduction in CH_4_ production by adding CH_4_ oxidizing bacterium isolated from the gut of young pigs. However, detailed in vivo studies are needed to establish the level of CH_4_ reduction. Another novel approach for enteric CH_4_ reduction is through the genetic modification of fermentation characteristics of rumen bacteria. However, research is still in the preliminary stages and very little progress has been made pertaining to applying the molecular techniques to characterize and quantify the microbial populations [86].

## 4. Conclusions and Future Perspectives

Goats undoubtedly need to be the priority focus for livestock industries due to their advantages over other ruminant animals from a climate resilience point of view. Elevated ambient temperature during the summer months can have a significant influence on the basic physiology of rumen, thereby affecting the rumen fermentation pattern, VFA and other rumen metabolites production. Furthermore, growing evidence suggests that heat stress influences the rumen microbial population, resulting in alterations in ruminal digestion process in goats. In addition, heat stress has also been shown to increase the production of enteric CH_4_ emission resulting in dietary energy loss. Thus, the productive performances of the animals are compromised. Nutritional interventions and other management strategies are traditional ways by which enteric CH_4_ emission is reduced in goats. More recently, several researchers have targeted reducing enteric CH_4_ through advanced biotechnological tools such immunization therapy, using bacteriocins, etc. but without much success. Further refinements in these technologies are essential before these technologies are implemented at field level. In the near future, these technologies offer scope for identifying genetically superior animals with less CH_4_ production per unit feed intake. However, further research efforts are needed to elucidate the mechanisms associated with enteric CH_4_ emission during heat stress exposure by establishing the relationships among the rumen microbes through metagenomics approaches in goats in the changing climate scenario. Such efforts may help to develop more focussed mitigation strategies for reducing enteric CH_4_ emission in goats. This may help to sustain goat production in the changing climate scenario by preventing the dietary energy loss incurred during the process of enteric CH_4_ emission.

## Figures and Tables

**Table 1:** Advantageous characteristics associated with goats over other livestock species to survive in harsh climatic conditions.

Criteria	Special Characteristics of Goats	References
Adaptability	Goats are better adapted to broad environmental conditions ranging from arid dry to cold arid to hot humid. Goats in the tropical warm climate are more or less dwarf and have less body weight, while goats in colder climates have bigger size and more fur growth. Due to their lesser body size, their metabolic requirements are considerably low, they have the ability to reduce their metabolism and their loose skin aids in easy dissipation of body heat.	[18]
Thermo-tolerance	Goats are more thermo-tolerant than all other ruminant species. They possess the ability to survive in different agro-ecological zones.	[19]
Drought tolerance	Goats possess the ability to thrive well in drought prone areas because of reduced water requirement in comparison to sheep and other domestic ruminants. Goats have better water conservation ability than other ruminant animals because of their browse diet. Further, the gut, especially the rumen, acts as a water reservoir during the periods of dehydration.	[19]
Ability to thrive well on low pasture	Efficient utilizers of poor quality and a wide range of pastures. Goats have improved digestibility compared to all other rumen and animals and, moreover, because the small-sized feed consumption is also low, these factors together favour less CH_4_ production.	[19]
Low enteric methane emission	Goats produce less enteric methane compared to sheep and other ruminants.	[20]
More demand for goat meat	Goat meat possesses less fat content and has no religious taboo; hence, it is relished by all. The lower saturated fat content in the goat meat improves the blood cholesterol level and stabilizes the heart rhythm of consumer. Goat meat contains vitamin B, B12 and omega-3 fatty acids. Further goat meat is lower in calories and cholesterol than the meat from other animals.	[21]
Milk with more nutrition	Goat milk is more nutritious than the milk from other species of livestock, easily digestible due to the presence of some beneficial fatty acids and contains fats and proteins in a finer state. Goat milk contains vitamin A, niacin, thiamin, ribofavin and pantotheanate.	[22]
Digestibility and feed conversion efficiency	Increased efficiency to convert feed into milk and meat than all other domestic ruminants, they can even digest poor quality feed. Goats have less proportion of gut in relation their total body weight, which enables the rapid movement of digesta from the rumen and the entire gastrointestinal tract.	[19]
Less initial investment	Minimum investment compared to large ruminants due to lower price. It is possible to get more animals at the cost of one cow. Less quantity of feed is required for goats compared to other domesticated livestock species.	[1]
Women entrepreneurship	Because of their small size, goats are easy to herd by women. They can let the animals graze on common property resources and private fallow lands. As they move as a herd, it is easy to track them.	[19]
Suitable for landless farmers	Small area is required to rear goats because of their small size, they require less feed and they can be easily integrated into other farming systems.	[14]

**Table 2:** Different impacts of heat stress on the rumen function in goats.

Type of Heat Stress	Effect on Rumen Fermentation Pattern	Reference
Summer heat stress	Altered basic physiology of rumen function	[23]
Extreme temperature stress	Reduced VFA production	[24]
Summer heat stress	Decreased rumen pH and acidosis	[25]
Heat stress	Reduction in ruminal pH; reduced rumen fermentation	[26]
Heat stress	Decreased rumen pH	[27]
Summer heat stress	Decreased VFA production; Reduced production of acetate	[28,29]
Heat stress	Decrease in acetate and acetate to propionate ratio and an increase in butyrate	[30]
Heat stress	Increase of *Streptococcus* genus bacteria and a decrease in the bacteria of *Fibrobactor* genus	[31]
Heat stress	Decrease in the *Streptococcus* genus and increase in *Clostridium coccoides–Eubacterium* genus	[32]
Increased temperature and RH	Decline in the concentrations of amylolytic and cellulolytic bacteria; decreased diet digestibility	[9]
Late summer	Increase in enteric CH_4_ emissions	[33]
Late summer season	Increase in enteric CH_4_ emissions	[34]
Summer heat stress	increase in CH_4_ emission	[35]

Note: RH: Relative humidity; VFA: Volatile fatty acid; CH_4_: Methane.
